# In Silico Analysis of Hepatitis B Virus Occult Associated Mutations in Botswana Using a Novel Algorithm

**DOI:** 10.3390/genes9090420

**Published:** 2018-08-21

**Authors:** Motswedi Anderson, Wonderful T. Choga, Sikhulile Moyo, Trevor Graham Bell, Tshepiso Mbangiwa, Bonolo B. Phinius, Lynette Bhebhe, Theresa K. Sebunya, Joseph Makhema, Richard Marlink, Anna Kramvis, Max Essex, Rosemary M. Musonda, Jason T. Blackard, Simani Gaseitsiwe

**Affiliations:** 1 Botswana Harvard AIDS Institute Partnership, Gaborone, Botswana; motswedi.anderson@gmail.com (M.A.); wtchoga@gmail.com (W.T.C.); smoyo@bhp.org.bw (S.M.); mbangiwat@gmail.com (T.M.); bphinius@gmail.com (B.B.P.); lynnettebhebhe@gmail.com (L.B.); jmakhema@bhp.org.bw (J.M.); rmarlink@globalhealth.rutgers.edu (R.M.); messex@hsph.harvard.edu (M.E.); rmusonda@bhp.org.bw (R.M.M.); 2 Faculty of Science, Department of Biological Sciences, University of Botswana, Gaborone, Botswana; sebunyat@gmail.com; 3 Department of Immunology and Infectious Diseases, Harvard T.H. Chan School of Public Health, Boston, MA 02115, USA; 4 Hepatitis Virus Diversity Research Unit (HVDRU), Faculty of Health Sciences, Department of Internal Medicine, School of Clinical Medicine, University of the Witwatersrand, Johannesburg 2050, South Africa; trevorgrahambell@gmail.com (T.G.B.); anna.kramvis@wits.ac.za (A.K.); 5 Faculty of Allied Health Sciences, University of Botswana, Gaborone, Botswana; 6 Rutgers Global Health Institute, Robert Wood Johnson Medical School, Rutgers University, New Brunswick, NJ 08854, USA; 7 College of Medicine, University of Cincinnati, Cincinnati, OH 45267, USA; blackajt@ucmail.uc.edu

**Keywords:** HBV, mutations, occult hepatitis B virus, in-silico analysis, Botswana, Hepatitis B, Africa

## Abstract

Occult hepatitis B infections (OBI) represent a reservoir of undiagnosed and untreated hepatitis B virus (HBV), hence the need to identify mutations that lead to this phenotype. Functionally characterizing these mutations by in vitro studies is time-consuming and expensive. To bridge this gap, in silico approaches, which predict the effect of amino acid (aa) variants on HBV protein function, are necessary. We developed an algorithm for determining the relevance of OBI-associated mutations using in silico approaches. A 3 kb fragment of subgenotypes A1 and D3 from 24 chronic HBV-infected (CHB) and 24 OBI participants was analyzed. To develop and validate the algorithm, the effects of 68 previously characterized occult-associated mutations were determined using three computational tools: PolyPhen2, SNAP2, and PROVEAN. The percentage of deleterious mutations (with impact on protein function) predicted were 52 (76.5%) by PolyPhen2, 55 (80.9%) by SNAP2, and 65 (95.6%) by PROVEAN. At least two tools correctly predicted 59 (86.8%) mutations as deleterious. To identify OBI-associated mutations exclusive to Botswana, study sequences were compared to CHB sequences from GenBank. Of the 43 OBI-associated mutations identified, 26 (60.5%) were predicted by at least two tools to have an impact on protein function. To our knowledge, this is the first study to use in silico approaches to determine the impact of OBI-associated mutations, thereby identifying potential candidates for functional analysis to facilitate mechanistic studies of the OBI phenotype.

## 1. Introduction

Occult hepatitis B infections (OBI) represent a significant reservoir of undiagnosed and untreated hepatitis B virus (HBV) infection. OBI is described as detectable HBV deoxyribonucleic acid (DNA) in the absence of detectable hepatitis B surface antigen (HBsAg) in the liver or serum [1,2]. OBI is characterized by very low viral loads, <200 IU/mL [2]. HBsAg negative infections with HBV DNA levels >200 IU/mL are deemed false OBI [2]. OBI prevalence ranges from 0% to 89.5%, although these cannot be compared directly because of differences in the sensitivity of laboratory tests used and testing algorithms [3,4,5,6,7,8,9]. In Botswana, HBsAg positivity rates ranging from 3.1% to 10.6% have been reported in Human Immunodeficiency Virus (HIV) positive individuals [10,11,12,13,14]. There is sparse data on the HIV negative group, with one study reporting a HBsAg positivity of 1.1% in HIV negative pregnant women [14]. On the other hand, OBI have been reported on 26.5% of HIV infected participants [15] and 5.7% of HIV positive pregnant women [14]. The latter study reported 7.4% OBI prevalence in HIV negative pregnant women [14]. The differences in prevalence might be due to differences in the cohorts and immune status of the participants. The clinical relevance of OBI has been demonstrated in several studies [16]. HBV from OBI can be transmitted through blood transfusions and solid organ transplantations resulting in either chronic or OBI [17,18,19,20]. OBI can also lead to serious clinical conditions such as hepatocellular carcinoma (HCC) and cirrhosis [21,22,23,24,25,26]. 

HBV, a DNA virus which belongs to the family *Hepadnaviridae*, replicates via an RNA intermediate [27,28]. The reverse transcriptase enzyme has no proof-reading capabilities, hence nucleotide misincorporation occurs during replication leading to sequence diversity [27,29]. HBV has been divided into at least nine genotypes (A-I) with a putative 10th genotype (J) [29,30]. The classification is based on nucleotide divergence of >7.5% at whole genome level [29,30]. These genotypes have been divided further into more than 35 subgenotypes based on the intragenotype divergence of 4–8% [29,31,32,33,34]. These genotypes, and in some cases subgenotypes, display a distinct geographic distribution, disease prognosis, and response to alpha interferon treatment [29,31,35]. Genotype recombination has been demonstrated between genotypes A/D, B/C, and C/D [36]. The circulating genotypes in Africa are A, D, and E. Similarly, genotypes A1, D3, and E have also been reported in Botswana [12,14,37]. 

There are several co-infections and mechanisms that may lead to the development of OBI [38], including coinfection with HIV [39,40,41] and hepatitis c virus (HCV) and multiple mutations in the HBV genome associated with OBI [42,43]. Even though a considerable number of OBI-associated mutations have been identified, functional studies on the consequences of these mutations are quite limited and those conducted to date focused primarily on the surface gene [16]. These studies have demonstrated that occult-associated mutations reduce HBV replication, increase retention of HBsAg within infected cells, alter post-transcriptional modification of HBsAg mRNA, and decrease the diagnostic ability to detect HBsAg [44,45,46,47,48,49,50,51,52]. In addition, studies of other HBV open reading frames (ORFs) have shown that the mutations and deletions are responsible for the OBI phenotype. For example, deletions in the basal core promoter region decrease replication and HBsAg expression [53] and may also affect the functions of HBx in the overlapping region. In the X region, these mutations may lead to a truncated HBx, thereby reducing the viral replication and secretion of HBsAg [54,55]. Mutations in the RT region of Pol may also affect the S gene as there is considerable overlap between these ORFs, causing a decrease in HBV replication competence, HBsAg secretion and antigenicity [56,57,58]. Several studies have identified mutations in the RNase H that also lead to a decrease in HBV replication [16].

In vitro functional analysis is the current gold standard for determining biological functions of proteins and their mutations. However, there are several bioinformatics tools that are used to predict such functions from amino acids (aa) or nucleotides [59,60] in silico. These include I-TASSER [61], SWISS MODEL [62], PSIRED [59], Phyre2 [63], and ROBETTA [64] server, which predict protein structure. The Phyre2 tool performs mutational analysis to determine whether an aa substitution has any impact on the biological function of the protein [63]. The Protein Variation Effect Analyzer (PROVEAN) also predicts aa substitutions, deletions, and insertions on the biological function of a protein [65]. Several of these tools were utilized in previous HBV studies but only to predict structures. I-TASSER was used to predict changes made by OBI-associated mutations on HBsAg structure [66], ROBETTA has been used to predict the effects of aa mutations on the structure of the S gene [67], while the MFOLD web server predicted the RNA secondary structure of a mutant OBI strain versus the wild strain around the 5’ splice site [68]. Bioinformatics tools use different designations to determine the impact of mutations on protein biological function. For example, PROVEAN classifies mutations with a negative impact on protein biological function as deleterious [65]. On the other hand, PolyPhen2 describes those with negative impact on protein function as damaging and those with no impact as benign [69], whereas SNAP2 uses ‘effect’ or ‘neutral’ to indicate the presence or absence of change in protein function caused by a mutation [70]. Despite their availability, these tools are rarely used to study HBV mutations [63,65]. Most HBV studies only predict structural changes caused by aa substitutions [66,67,68]. Nonetheless, a number of HBV mutational studies have been published recently, and most report the existence of occult-associated mutations without conducting any functional characterization either in vitro or in silico [42,43,44,71]. For example, a total of 235 OBI-associated mutations were reported [42] but only 7 were characterized in subsequent in vitro analysis. Thus, there is a need to bridge the gap between HBV mutational studies and in vitro functional studies by identifying the best possible candidates for subsequent functional analysis using robust bioinformatics tools.

Many OBI mutations have been identified, although only very few have been functionally characterized, and there are no data regarding the use of these bioinformatics tools to identify OBI-associated mutations for additional functional analysis. Here, we developed an algorithm for determining the relevance of some occult associated mutations in the OBI phenotype using an in silico approach and tested the use of these tools in OBI-associated mutations identified in HBV strains from Botswana.

## 2. Materials and Methods

### 2.1. Population

Nearly whole genome sequences (3 kb) of HBV isolated from 24 CHB and 24 OBI participants were utilized for this study. The isolates were from baseline samples from two previous studies conducted at Botswana Harvard AIDS Institute Partnership, Gaborone, Botswana: The Botswana National Evaluation Models of HIV Care (Bomolemo study) and The Effects of HIV and ARV Exposure on Child Health and Neurodevelopment (Tshipidi study), which were conducted between 2009 and 2012 [15,72]. The 48 sequences included 12 subgenotype A1 and 12 subgenotype D3 samples per group (CHB and OBI).

### 2.2. Determination of OBI-Associated Mutations

The sequencing of the HBV genomes was reported in detail elsewhere [73]. Briefly, nearly whole HBV genome was successfully sequenced from 50 of 109 participants (37 CHB and 72 OBI positive) using big dye sequencing chemistry. The nearly whole genome fragment (3 kb excluding the precore region) was amplified by nested PCR [73]. Online databases were used to determine the genotypes; Geno2pheno available at https://hbv.geno2pheno.org/ and Stanford HBV database available at https://hivdb.stanford.edu/HBV/HBVseq/development/HBVseq.html. To confirm the genotypes, phylogenetic trees were constructed utilizing a Bayesian Markov Chain Monte Carlo (MCMC) in the Bayesian Evolutionary Analysis by Sampling Trees (BEAST) v1.8.2 program with a chain length of 100,000,000 and sampling at every 10,000th generation [74]. Simplot software version 3.5.1 was used to check for recombination [75]. None of the sequences were identified as intergenotypic recombinants.

### 2.3. Ethical Considerations

The study was approved by University of Botswana Institute Review Board and the Health Research Development Committee (HRDC) at the Botswana Ministry of Health and Wellness.

### 2.4. In Silico Methods

Three programs were used to determine if OBI mutations have any impact on the biological function of the protein: The Protein Variation Effect Analyzer (PROVEAN) [65], PolyPhen2 [69], and Screening for Non-Acceptable Polymorphisms (SNAP2) [70] programs. Phyre2 was used to depict the positions of OBI-associated mutations in the core region [63]. Briefly, PROVEAN is a bioinformatics tool utilizing an alignment-based score to predict the functional deleterious effects of a single aa mutation, multiple aa mutations, deletions, as well as insertions in both human and non-human proteins [65]. Alignment scores are used to gauge sequence similarity in pairwise sequence alignments. An alteration in the alignment score caused by a mutation corresponds to the impact of that mutation in the function of the protein. Results are described as either deleterious (negative impact) or neutral. The PROVEAN program process is freely available at http://PROVEAN.jcvi.org [65]. 

PolyPhen-2 is a method for predicting the effects of human missense mutations and is available at http://genetics.bwh.harvard.edu/pph2/. This method uses a self-operating sequence alignment procedure in which the selected homologs and the calibre of the multiple sequence alignment (MSA) have a major impact on the final results. In brief, after user input, (non-synonymous single nucleotide polymorphism (nsSNP) and protein accession or sequence) homologues are searched by BLAST+ and aligned utilizing MAFFT, the alignment is then refined by Leon software to remove substandard parts. The Secator algorithm administered in ClusPack software is ultimately employed to cluster the now quality aligned sequences [69]. Results are described as ‘damaging’, ‘possibly damaging’, ‘probably damaging’, and ‘benign’ indicating varying degrees of the negative impact and lack of impact on protein function by the mutation.

The SNAP2 is a method which can differentiate neutral aa substitutions from those that have an effect in both human and non-human proteins [70]. It uses aa features, predicted role, and the structure of the protein to predict the effect of mutations [70]. However, SNAP2 can predict the effects even without the structure and it can forgo the use of evolutionary information in its prediction. The results span a range of −100 to +100 depicting a strongly neutral to a strongly predicted effect of variants respectively [70]. Results are described as ‘effect’ or ‘neutral’ indicating the absence or presence of change in molecular function caused by a mutation. SNAP2 is freely available at http://www.rostlab.org/services/SNAP2. 

### 2.5. Data Analysis

A total of 48 whole genome/nearly full-length sequences were aligned with their corresponding subgenotype references from GenBank using ClustalX software version 2.1 [76]. The reference sequences included were all HBV whole genome sequences from HBsAg positive individuals compiled from http://hvdr.bioinf.wits.ac.za/alignments [77]. Additional information regarding the HBsAg status of the individual from which the sequence was derived was obtained from GenBank entries and the original publications. The 24 subgenotype A1 sequences from Botswana were aligned with 107 full-length subgenotype A1 references. The 24 subgenotype D3 sequences from Botswana were aligned with 85 subgenotype D3 full-length reference sequences from GenBank. Sequences were then trimmed to the same length using BioEdit version 7.2.5. Subsequently, the Babylon Translator tool [78] was utilized to extract each protein and translate the sequences into amino acid regions corresponding to the Pre S1, Pre S2, S, X, PreC, core, or Pol region. The HBV sequences isolated from OBI participants were first compared with those from CHB positive participants for each respective subgenotype and then compared to the reference sequences (references from other parts of the world followed by those from South Africa). Mutations that were unique to HBV isolated from OBI participants without appearing in any sequences from CHB participants or GenBank references from CHB were classified as OBI-associated mutations [42].

PROVEAN, PolyPhen2, and SNAP2 were used to predict whether the OBI-associated mutations have any impact on the biological function of the viral protein. To identify the best combination of these tools to use for final result interpretation, 68 mutations, previously described in the literature, that have been functionally characterized and known to have a deleterious effect in the S region of HBV, were used [16]. These mutations were tested *in silico* in the same genotype background as the one used in the respective in vitro studies. Most studies only mentioned the serotype of the genetic background used in the functional studies. However, some serotypes like *adw*2 (genotypes A, B, G, I) are linked to multiple genotypes [79]. It was important to test the mutation in the correct background as the same mutation might have a different effect across distinct genotypes [49,80]. Depending on the input requirements, a wild type consensus sequence and the mutations (PROVEAN and PolyPhen2) or just a wild type consensus sequence (SNAP2) were submitted to the three bioinformatics tools [65,69,70]. From a wild type consensus sequence, SNAP2 provides the impact of other aa in all positions (all possible results combinations) [70]. The results of the prediction tools were compared, and the mutation was considered as correctly predicted if noted as deleterious by at least two of the three prediction tools. Damaging, possibly damaging, and probably damaging results from PolyPhen2 were changed to deleterious, whereas benign was changed to neutral for uniformity in reporting results for the three tools. In SPAN2, the result ‘Effect’ was changed to deleterious also for uniformity of results between the three in silico tools. Phyre2 was used to show positions of study mutations on C region structure.

## 3. Results

The baseline demographics have been compared in detail elsewhere. There was no statistically significant difference in the baseline samples in CD4+ T cell counts, HIV viral load, liver enzymes, FIB4, and other clinical parameters based on HBsAg status [14,15]. The HBV viral loads were low in the OBI group with the median of 57.4 copies per mL versus 3.1600 copies per mL in the CHB group and 68.1% of OBI patients having HBV viral loads <116.4 copies per mL as reported elsewhere [15].

**Figure 1:**
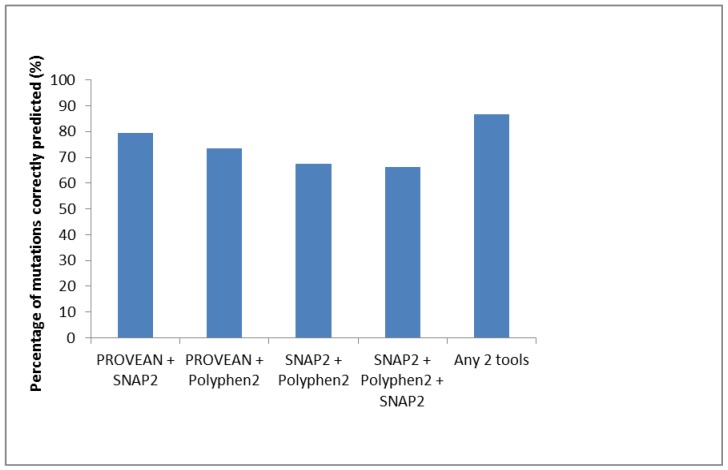
Percentage of mutations previously shown to be deleterious that were identified by the various prediction tools.

PolyPhen2 and SNAP2 were used to predict the effects of the 68 functionally characterized mutations, located within the surface region that were known to be deleterious based on the available literature. These tools detected 52 mutations (76.5%) as possibly or probably having a negative impact and 55 (80.9%) as altering the molecular function of the protein, respectively. PROVEAN also predicted 52 mutations (76.5%) as deleterious at the default cut-off of −2.5 but detected 65 (95.6%) at the lowest cut-off allowed by the assay (−1.300). When combining the three prediction tools (−2.5 cut-off for PROVEAN), 34 mutations were predicted as having an effect by all three tools, whereas a further 22 were predicted by at least two tools. Collectively, 56 mutations (82.4%) were correctly predicted as having an effect on protein function by at least two tools. Three mutations—sA159G, sI126S, and sL98V—were not identified by any of the prediction tools. On the other hand, at a PROVEAN cut-off of −1.3, 46 mutations were correctly predicted by all three tools as deleterious, while a further 13 were predicted by at least two tools. Thus, 59 (86.8%) were correctly detected (Table 1, Figure 1). Only 1 mutation (sI126S) was predicted as neutral by all three tools. PolyPhen2 gives specificity for each result and all values were ≥78%, whereas the average of expected accuracy for SNAP2 results was 73%. The accuracy for PROVEAN was found to be >73% when supporting sequences used were ≥50 [65], and >50 homologues were used for all the predicted results in this study. Two additional tools were tested but found to not be appropriate (data not shown). The ‘Sorting Tolerant From Intolerant’ (SIFT) tool was used to analyze the mutations, but it returned predictions with very low confidence, stating that the sequences used to make the prediction ‘might not be diverse enough’. Phyre2 can also perform mutational analysis, but it failed to analyze the HBV ORFs with the exception of the core region.

The three prediction tools were also used to predict mutations that were functionally characterized and found to be neutral in literature. PROVEAN predicted 18 out of 32 mutations (56.3%) to be neutral at a cutoff of −2.5, whereas 8 (25%) were neutral at a cutoff of −1.3. PolyPhen2 and SPAN2 predicted 23 (71.9%) and 11 (34.4%) as neutral, respectively. In total, 16 (50%) and 10 (31.3%) mutations were predicted by at least two prediction tools as neutral at PROVEAN cut off −2.5 and −1.3. (Table 2). The prediction scores on the neutral aa variants were low because it depends on the assays, which were used and most of the times studies are not exhaustive. 

### OBI-Associated Mutations Phenotypic Results

The algorithm developed above was used to determine the impact of 43 OBI-associated mutations from Botswana, which have been described in detail elsewhere [73]. Several of these mutations have been characterized in vitro previously, while others have been reported but not functionally characterized in vitro. Twenty-six were novel mutations that had never been reported nor characterized previously. Each of the OBI associated mutations was found in only one of the OBI sequences (Table 3). The 6 OBI-associated mutations in the S region, 2 in the PreS2 region, and 1 in the PreS1 region were all predicted to be deleterious by at least two prediction tools. Similarly, 2 of the 7 OBI-associated mutations in the X region, 4 out of 12 in the Pol region, and 11 out 15 mutations in the core region were predicted as deleterious (Table 3).

#### Phyre2 Results

**Figure 2:**
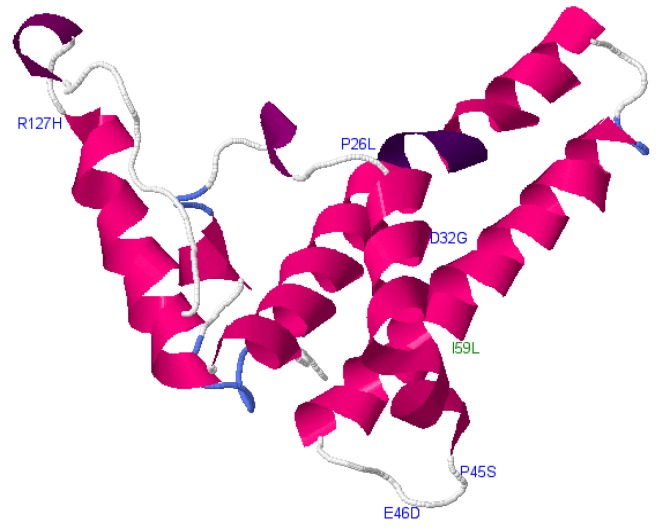
Mutations within the core region of subgenotype A1.

**Figure 3:**
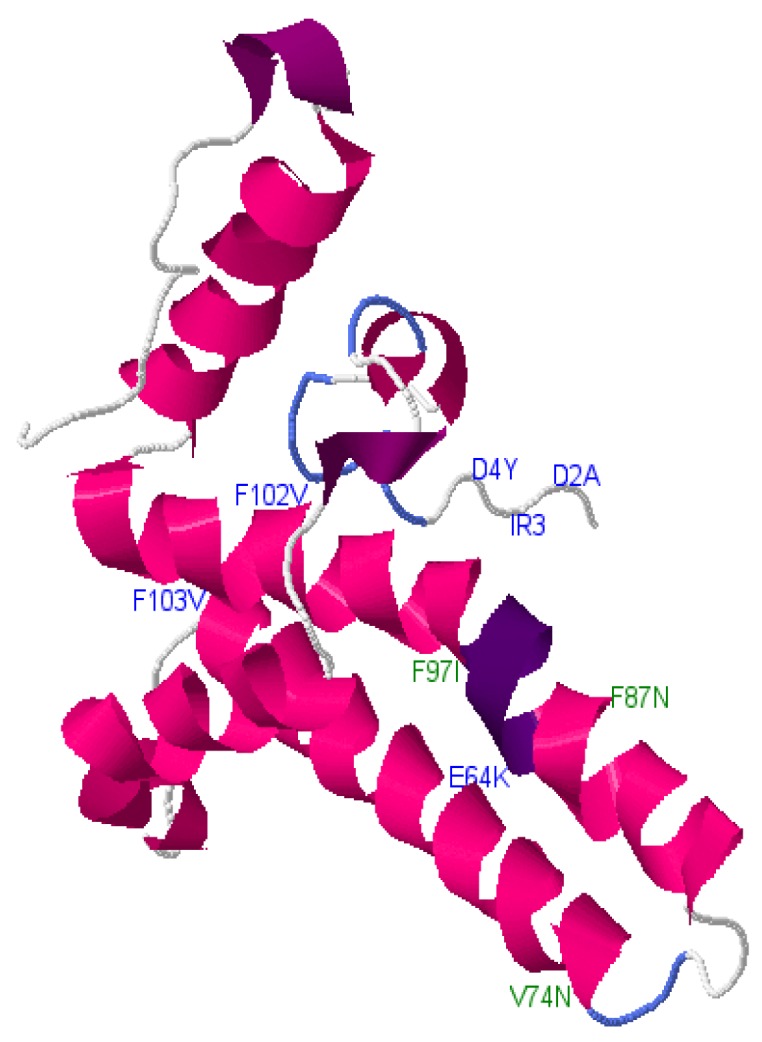
Mutations within the core region of subgenotype D3.

Phyre2 was used to illustrate mutation positions within the core structure (Figure 2 and Figure 3). This was done to determine whether OBI-associated mutations from this study were concentrated in certain areas in the protein structure. Core was chosen for this analysis because Phyre2 was able to predict its structure as opposed to the other reading frames for which no structure was available or the confidence of the prediction was too low.

The core-protein-predicted 3-dimensional structure was generated using Phyre2 and viewed using JSmol interactive viewer. Positions corresponding to the mutations from this study are labelled using the wild type amino acid (designated by a letter) and the number represents the position of the mutation. Deleterious mutations are labelled in blue whereas the neutral ones are in green.

The core-protein-predicted 3-dimensional structure was generated using Phyre2 and viewed using JSmol interactive viewer. Positions corresponding to the mutations from this study are labelled using the wild type amino acid (designated by a letter) and the number represents the position of the mutation.

## 4. Discussion

Multiple OBI-associated mutations have been identified in several studies. Powell et al*.* identified 235 OBI-associated mutations in South Africa [42]. However, the functional studies required to elucidate the effects of these mutations in the viral phenotype are both expensive and time-consuming [91]. Identifying potential candidate mutations with possible functional relevance is necessary to reduce the number included in in vitro studies. 

In silico analyzes have been used to predict disease-causing single nucleotide variants in humans, and their use has been extended to other organisms. For example, Dakal et al*.* used PROVEAN, PolyPhen2, and SIFT to predict the functional effects of aa variants in the interleukin 8 gene [92], whereas Desai et al. utilized the same tools to identify deleterious variants in methylenetetrahydrofolate dehydrogenase 1 [93]. The prediction tool predicts whether the aa variant is likely to have an effect in protein function or not [65,69,94]. However, to date this approach has not been utilized for studies of HBV isolated from occult HBV infection.

To the best of our knowledge, this study represents the first in silico analysis of OBI-associated mutations conducted to identify possible candidates for functional studies. Using three prediction tools, 86.8% of aa variants that have been functionally characterized and confirmed to have an impact in HBV surface gene function were correctly predicted by at least two of these tools. A combination of in silico approaches has been used to develop algorithms for phenotype predictions. Ou et al*.* used a combination of bioinformatics tools and achieved 94% sensitivity in identifying potential single aa variants with functional relevance in investigating mucopolysaccharidosis type I disease [95]. In our study, PolyPhen2 detected 76.5% of the deleterious mutations; Sadowski et al. reported 88.9% in the categorization of aa variants in BRCA1/2 [96]. The difference may be attributable to the fact that the latter study conducted a prediction in human genetics and these tools perform better as they were originally designed to predict disease causing variants in humans [69]. On the other hand, PROVEAN and SNAP2 were designed to also predict the impact of mutations in non-human sequences [65,70]. The sI126S mutation was not detected by all three tools as deleterious even though it has been shown to reduce extracellular HBsAg and reduce viral secretion in vitro and in vivo*.* [49]. These results indicate that in silico approaches can also be used to predict potential candidates for functional analysis of OBI-associated mutations. The algorithm was also employed in neutral mutations but did not perform as well as in deleterious mutations. This might be because it is difficult to find functionally characterized neutral HBV mutations in the literature as some mutations might be reported as having no effect in biological function of the protein just because certain aspects where not tested. For example, one study reported **s**P120T [87] as neutral on genotype C, but it was found to decrease HBsAg antigenicity and extracellular HBsAg by two other studies using both genotype B and C [49,81]. **s**G145R and **s**R160G were also reported as having no impact on genotype C [87]; yet, multiple studies associate it with decreased HBsAg antigenicity, immunogenicity, extracellular HBsAg, and viral secretion on genotypes A–C [49,50,81,83,86,97]. In fact, sG145R is the classical HBsAg detection escape mutant.

In the current study, we report for the first time the presence of OBI-associated mutations with potential effects on protein function in HBV isolated from Botswana. Of the 43 OBI-associated mutations identified in this study, 26 were predicted to have an impact on protein function. Most of the predicted deleterious OBI-associated mutations appeared in the surface and core regions. Several mutations in the core region were located in functionally relevant regions such as the CD4^+^ T cell epitopes (cD2A, cV13R, cD4Y, cE64K, and cR127H) [98]. 

In summary, an in silico approach has been used for the first time to predict OBI-associated mutations that have an impact on protein function. This strategy will allow for the identification of possible candidates for in vitro functional analysis in OBI studies. There are many OBI-associated mutations that have been identified, and it might be expensive and time consuming to functionally characterize them all. Some of the OBI-associated mutations reported in the literature might just be lineage specific polymorphisms [16,31]. Some of the OBI-associated mutations from Botswana were predicted as having an impact on protein function; hence, they may account for phenotypes associated with OBI, including undetectable HBsAg and low HBV viral loads. Some of the deleterious mutations might be affecting protein function but not necessarily leading to the OBI phenotype.

A limitation of this study is that the prediction tools were evaluated using a limited set of OBI-associated mutations because there are very few functionally characterized OBI-associated mutations compared to characterized human disease-causing variants. Another limitation was that there are few neutral OBI-associated variants because studies are not exhaustive (a mutation may be reported as neutral because other aspects were not studied). The variant is usually regarded as having no impact because of the limitations of the assays performed or because phenotypic systems are in isolation hence might not detect the impact of some compensatory mutations. The other limitation is that the tools used could only predict protein changes that may have an effect on protein function. However, OBI can also be the result of mutations acting at the transcriptional level. Furthermore in vitro studies have shown that mutations can work together to change the protein function and some mutations can reverse the effects of other mutations; however, the effects of multiple mutations were not tested in this study as only PROVEAN could analyze multiple mutations. Hence, even mutations classified as neutral might have an impact when in combination with other mutations. Additionally, the impact of mutations in overlapping regions like RT and S could not be analyzed. Lastly, a limited number of prediction tools were used as some of the other tools could not give a prediction or the confidence of the prediction was too low. Discrimination between diagnostic OBI and true OBI was not performed because most of the OBI participants had HBV viral loads <200 IU/mL with only 6 out of 72 having viral loads >200 IU/mL. Population sequencing was used to generate the sequences, in cases with multiple nucleotides occurring at a frequency of 20% or above in the same position, ambiguity codes were used to cater for the polymorphism. We cannot rule out the presence of variants at frequencies of less than 20%, which would usually not be picked by populations sequencing.

Future studies employing more in silico mutation prediction tools to predict the impact of OBI-associated mutations are crucial and are a necessity. The structural-based prediction tools were unable to predict the effects of several mutations because of the lack of homology structures in the database. Also, functional analysis studies should be performed on the OBI-associated mutations that were predicted as having deleterious impacts on protein function in order to elucidate how they affect protein function and contribute to the OBI phenotype.

## Figures and Tables

**Table 1:** Predicted effects of functionally characterized deleterious mutations of the surface region of hepatitis B virus (HBV) from literature.

Variant	Genotype	PROVEAN Prediction ^#^	PROVEAN Prediction *	SNAP2 Prediction	PolyPhen2	References
sN146D, sC147Y, sY100C, sG130R, sC138Y, sP142S, sG145R, sG145E, sN146S, sC147R, sC149Y, sD144G	A	✔	✔	✔	✔	[81,82]
sS132P, sT114R, sT115A, sK141E	A		✔	✔	✔	[71,81]
sC121A, sC124A, sQ129H, sC147A	A	✔	✔	✔		[81]
sP120T	A	✔	✔		✔	[81]
sP127S	A		✔		✔	[81]
sC149A	A	✔	✔			[81]
sC124R, sS136P, sC139R, sT140I, sD144A, sG145R, sG145A, sG145W, sG145I, sG145P, sG145N, sG145D, sK122I	B	✔	✔	✔	✔	[49] [83] [61]
sK141E, sK160N, sT123N	B		✔	✔	✔	[49,84]
sQ129R	B		✔			[49]
sP120T	B	✔	✔		✔	[49]
sA159G	B				✔	[84]
sG119R, sC124R, sC124Y, sS136P, sC139R, sK141E, sL21R, sT131I, sP142S	C	✔	✔	✔	✔	[49] [85] [86]
sG145R, sG145A	C	✔	✔	✔		[86]
sL95W	C		✔	✔	✔	[85]
sD144A	C	✔	✔		✔	[49]
sT140I, sP120T, sE2G	C	✔	✔			[49] [85]
sL98V	C			✔	✔	[85]
sD99G	C		✔	✔	✔	[87]
sQ129R	C		✔			[49]
sM133T	C		✔	✔		[88]
sI126S	C					[49]
sT116N	D		✔	✔	✔	[66]
sP120T	D	✔	✔		✔	[80]
sR122P, sG145R	D	✔	✔	✔		[66] [85]
sT125M	D	✔	✔	✔	✔	[89]
Total (Effect)		52 (76.5%)	65 (95.6%)	55(80.1%)	52 (76.5%)	
Total (Neutral)		16	3	13	16	

S: Surface: ✔ Indicates an effect in protein function; empty cells denotes a neutral effect of the aa variant; **^#^**cut-off = −2.5; * cut-off = −1.3.

**Table 2:** Predicted effects of functionally characterized neutral mutations of HBV from literature.

Variant	Region	Genotype	PROVEAN Prediction ^#^	PROVEAN Prediction *	SNAP2	PolyPhen2	Reference
sM103I	S	A		✔	✔		[44]
sK122R	S	A					[44]
**tp**P100S	TP	A	✔	✔	✔		[45]
sP111S, sS154P, sK122P, sK122W	S	B	✔	✔	✔	✔	[83,90]
sG112E, sG119E, sW165R, sK122G, sK122L, sK122D	S	B	✔	✔	✔		[83,90]
sQ129R	S	B		✔			[90]
sI150T	S	B	✔	✔		✔	[90]
sK122M, sK122H	S	B		✔	✔	✔	[83]
sK122Q	S	B					[83]
sK122T, sK122E, sK122N	S	B		✔	✔		[83]
**tp**Q177H	TP	B				✔	[45]
**tp**R27L	TP	B		✔	✔		[45]
sC121Y	S	C	✔	✔	✔	✔	[87]
sR24K, sT47A, sT47K, sI126S, sF134Y	S	C					[85,86,87]
sQ101R	S	D		✔			[66]
sS167L	S	D	✔	✔	✔		[66]
sS143L	S	D		✔	✔		[66]
total			18 (56.3%)	8 (25%)	11 (34.4%)	23 (71.9%)	

S: Surface; TP: Terminal protein; ✔ Indicates an effect in protein function; empty cells denotes a neutral effect of the aa variant; **^#^**cut-off = −2.5; * cut-off = −1.3.

**Table 3:** Results of predicted effects of study occult hepatitis B infection (OBI)-associated mutations on different regions of HBV.

Variant	ORF	Genotype	Final Result
sL97P, sT114I, sC124Y *, sN131K *, sP217L *	S	A1	Deleterious
sQ129H *	S	D3	Deleterious
**_PreS1_S78N**	PreS1	D3	Deleterious
**_PreS2_F22P**, **_PreS2_F22H**	PreS2	D3	Deleterious
**xS11A**, **xV15I**	X	A1	Neutral
xS31A, xS101P, xL116V	X	D3	Neutral
**xP11S**, xQ87L	X	D3	Deleterious
**cS26P**, **cD32G**, **cP45S**, cE46D, cR127H	Core	A1	Deleterious
**cI59L**	Core	A1	Neutral
cV74N, cS87N, cF97I	Core	D3	Neutral
**cD2A**, **D4Y**, **cI3R**, cE64K, **cW102V**, **cF103V**	Core	D3	Deleterious
**tpN120Y**, **tpK155R**	Pol-TP	A1	Deleterious
**spS91T**, **spS133G**	Pol-Spacer	A1	Neutral
rtL140I, **rtA329T**	Pol-RT	A1	Neutral
**rtT225A**	Pol-RT	A1	Deleterious
**rhI81M**	Pol-RH	A1	Neutral
**spW64R**, **spP103S**	Pol-Spacer	D3	Neutral
**rtY257F**	Pol-RT	D3	Neutral
rtT128I	Pol-RT	D3	Deleterious

*: Functionally characterized in literature and found to affect OBI phenotype. Novel OBI-associated mutations are shown in bold. Non-bold non-asterisk means mutations reported in literature but not functionally characterized in vitro. ORF: Open reading frame; S: surface; PreS1: Pre Surface 1, PreS2: Pre Surface 2 Pol: Polymerase, TP: Terminal protein, RT: Reverse transcriptase, RH: RNase H.
